# Infection Strategies Deployed by *Botrytis cinerea*, *Fusarium acuminatum*, and *Rhizopus stolonifer* as a Function of Tomato Fruit Ripening Stage

**DOI:** 10.3389/fpls.2019.00223

**Published:** 2019-03-01

**Authors:** Stefan Petrasch, Christian J. Silva, Saskia D. Mesquida-Pesci, Karina Gallegos, Casper van den Abeele, Victor Papin, Francisco J. Fernandez-Acero, Steven J. Knapp, Barbara Blanco-Ulate

**Affiliations:** ^1^Department of Plant Sciences, University of California, Davis, Davis, CA, United States; ^2^Laboratory of Phytopathology, Wageningen University, Wageningen, Netherlands; ^3^Laboratory of Plant Physiology, Wageningen University, Wageningen, Netherlands; ^4^Ecole Nationale Supérieure Agronomique de Toulouse, Toulouse, France; ^5^Microbiology Laboratory, Institute of Viticulture and Agri-Food Research, Marine and Environmental Sciences Faculty, University of Cádiz, Cádiz, Spain

**Keywords:** broad host range pathogens, necrotrophic fungi, fruit-pathogen interactions, rotting, necrotic response, cell wall degrading enzymes, redox, *de novo* transcriptomes

## Abstract

Worldwide, 20–25% of all harvested fruit and vegetables are lost annually in the field and throughout the postharvest supply chain due to rotting by fungal pathogens. Most postharvest pathogens exhibit necrotrophic or saprotrophic lifestyles, resulting in decomposition of the host tissues and loss of marketable commodities. Necrotrophic fungi can readily infect ripe fruit leading to the rapid establishment of disease symptoms. However, these pathogens generally fail to infect unripe fruit or remain quiescent until host conditions stimulate a successful infection. Previous research on infections of fruit has mainly been focused on the host’s genetic and physicochemical factors that inhibit or promote disease. Here, we investigated if fruit pathogens can modify their own infection strategies in response to the ripening stage of the host. To test this hypothesis, we profiled global gene expression of three fungal pathogens that display necrotrophic behavior—*Botrytis cinerea*, *Fusarium acuminatum*, and *Rhizopus stolonifer*—during interactions with unripe and ripe tomato fruit. We assembled and functionally annotated the transcriptomes of *F. acuminatum* and *R. stolonifer* as no genomic resources were available. Then, we conducted differential gene expression analysis to compare each pathogen during inoculations versus *in vitro* conditions. Through characterizing patterns of overrepresented pathogenicity and virulence functions (e.g., phytotoxin production, cell wall degradation, and proteolysis) among the differentially expressed genes, we were able to determine shared strategies among the three fungi during infections of compatible (ripe) and incompatible (unripe) fruit tissues. Though each pathogen’s strategy differed in the details, interactions with unripe fruit were commonly characterized by an emphasis on the degradation of cell wall components, particularly pectin, while colonization of ripe fruit featured more heavily redox processes, proteolysis, metabolism of simple sugars, and chitin biosynthesis. Furthermore, we determined that the three fungi were unable to infect fruit from the *non-ripening* (*nor*) tomato mutant, confirming that to cause disease, these pathogens require the host tissues to undergo specific ripening processes. By enabling a better understanding of fungal necrotrophic infection strategies, we move closer to generating accurate models of fruit diseases and the development of early detection tools and effective management strategies.

## Introduction

Fungi are important plant pathogens that cause large economic losses due to their ability to inflict diseases such as rot, rust, and wilt in various plant organs both preharvest and postharvest (Dean et al., 2012; Nabi et al., 2017). Biotrophic fungi feed on living cells and suppress the host immune system by secreting effector proteins (Oliver and Ipcho, 2004; Glazebrook, 2005). In contrast, necrotrophic fungi feed on dead host cells and cause necrosis by secreting toxins and cell wall-degrading enzymes (CWDEs), among other virulence factors (van Kan, 2006). Due to their ability to feed on dead host tissue, necrotrophic fungi are also sometimes grouped into the less defined group of saprotrophic fungi, which includes many fungi that do not actively kill host cells (Dyakov, 2007). Additionally, hemibiotrophs are pathogens that start their infection cycle as biotrophs and end as necrotrophs (Perfect and Green, 2001). Biotrophic infection mechanisms are well-studied, whereas those of necrotrophic fungi are less understood. The lower scientific interest in necrotrophic infection mechanisms may be due to their perceived lack of specificity. The brute force strategy of secreting toxins and CWDEs as well as the broad host range of many necrotrophic fungi is often interpreted as indiscriminate killing of host cells without the requirement for host-pathogen compatibility (van Kan, 2006). However, the reality of necrotrophic infections is multifaceted, as they involve several features initially believed to be unique to biotrophs, e.g., the suppression of the host immune system or symptomless endophytic growth (van Kan et al., 2014; Veloso and van Kan, 2018). The relevance of host-pathogen compatibility in necrotrophic infections is also highlighted by the fact that necrotrophic fungi can readily infect ripe fruit but fail to infect unripe fruit or remain quiescent until host and environmental conditions stimulate a successful infection (Prusky and Lichter, 2007; Alkan and Fortes, 2015; Blanco-Ulate et al., 2016a,b).

To develop a better understanding of how fungi attempt to establish infections in fruit, we studied three impactful pathogens with broad host range: *Botrytis cinerea*, *Fusarium acuminatum*, and *Rhizopus stolonifer*. *B. cinerea* is the causal agent of gray mold, an economically devastating disease, and serves as a model species for plant-necrotroph interactions (Fillinger and Elad, 2016). In compatible hosts, such as ripe fruit, *B. cinerea* produces toxins, CWDEs, reactive oxygen species (ROS), and other virulence factors to induce rapid death and decay of the plant tissues (Nakajima and Akutsu, 2014; Blanco-Ulate et al., 2016a). In incompatible hosts, such as unripe fruit, *B. cinerea* establishes quiescent infections while suppressing the host immune system and promoting susceptibility in the host (Prusky et al., 2013; Weiberg et al., 2013). *B. cinerea* has been shown to activate fruit ripening processes, including changes in plant hormone biosynthesis and signaling and induction of host CWDEs involved in fruit softening, all of which seem to favor fungal growth and colonization (Swartzberg et al., 2008; Cantu et al., 2009; Blanco-Ulate et al., 2013). Even though *B. cinerea* infection strategies have been studied in various pathosystems (Ferrari et al., 2007; Cantu et al., 2009; Rossi et al., 2017), it is mostly unknown whether *F. acuminatum* and *R. stolonifer*, two understudied fungal pathogens, implement similar mechanisms when interacting with compatible and incompatible hosts. *F. acuminatum* has been reported to infect roots and fruit (Visconti et al., 1989; Logrieco et al., 1992; Jiménez et al., 1993; Marín et al., 2012). Within the *Fusarium* genus, *F. acuminatum* is among the most toxic species as it produces strong mycotoxins, such as trichothecene and fumonisins, to kill host cells and induce tissue necrosis (Visconti et al., 1989; Altomare et al., 1997). *R. stolonifer* causes rotting of fruit and other fresh products, mainly by secreting CWDEs, and is considered to be one of the most destructive postharvest pathogens due to its extremely fast growth rate (Bautista-Baños et al., 2008, 2014).

We leveraged the fact that tomato fruit display an increase in susceptibility to necrotrophic fungal infection as a result of ripening to develop a system for studying compatible and incompatible host-pathogen interactions. The transition from unripe to ripe fruit results in a markedly different physicochemical environment for colonization. In comparison to unripe fruit, ripe fruit have higher levels of total soluble solids, greater titratable acidity (TA), lower firmness, and a different composition of secondary metabolites and volatiles (Blanco-Ulate et al., 2016b). In light of this, we anticipated that these pathogens would exhibit specific patterns of gene expression based on the fruit ripening stage and that the functions of these genes would reflect important strategies for interaction with the different host environments. First, we evaluated the incidence and progression of fungal infections caused by *B. cinerea*, *F. acuminatum*, and *R. stolonifer* when inoculated in tomato fruit. Then, to determine if the pathogens adapted their infection strategies as a function of the host developmental stage, we analyzed the transcriptomes of each fungus at two points post-inoculation in unripe and ripe tomato fruit and compared these against their transcriptomes when grown under *in vitro* conditions. This approach allowed us to identify specific pathogenicity and virulence factors, e.g., CWDEs and toxin biosynthetic genes, that are differentially or commonly deployed by the pathogens in each host tissue. As necrotrophic infection strategies may be evolutionarily conserved as well as highly specific, we used the transcriptomic data to examine virulence functions among the three fungi and identified similarities in the adaptations of the pathogens to the different environments of ripe and unripe fruit. Finally, to further validate necrotrophic strategies dependent on the ripening stage of the fruit host, we evaluated the pathogenicity of the three fungal pathogens in fruit in a non-ripening tomato mutant. Overall, the approach followed in this study provides an initial platform to perform comparative transcriptomics among three fungi that cause economically relevant fruit diseases and sheds light into how pathogens with necrotrophic lifestyles adapt their infection mechanisms during compatible and incompatible interactions.

## Materials and Methods

### Plant and Fungal Material

Tomato fruit (*Solanum lycopersicum*) from the cultivar Ailsa Craig (AC) and the isogenic mutant *non-ripening* (*nor*) were used in this study. Plants were grown under field conditions in Davis, CA, United States, during the 2017 season. Mature green (MG) fruit were harvested 31 days post-anthesis (dpa) and red ripe (RR, or equivalent RR-like for *nor*) fruit at 42 dpa. The fungal pathogens studied were *B. cinerea* strain B05.10, an isolate of *R. stolonifer*, and an isolate of *F. acuminatum*. The isolates of *R. stolonifer* and *F. acuminatum* were obtained from postharvest infections of fresh produce and identified using morphological and sequencing methods. All fungi were grown on 1% potato dextrose agar (PDA) plates at room temperature (RT) until sporulation. Spore suspensions were prepared in 0.01% Tween^®^ 20 (Sigma-Aldrich, United States). Fungi from axenic *in vitro* cultures were grown on 1% PDA plates at RT, and mycelium for RNA extraction was harvested before the fungi reached the sporulation stage.

### Fruit Inoculations

Tomato fruit from AC and *nor* were collected at MG and RR or RR-like stage. Selected AC MG fruit were green, firm, and had soluble solids content (SSC) of 5.24 ± 0.44 g sucrose/100 g solution and TA of 5.20 ± 1.24%. AC RR fruit were bright red, pliable when squeezed, and had SSC of 6.27 ± 0.42 g sucrose/100 g solution and a TA of 3.47 ± 0.26%. *nor* MG fruit were similar to AC MG fruit, and *nor* RR-like fruit were selected that were green in color slightly soft at the blossom end. Fruit were sterilized in 0.6% sodium hypochlorite, wounded four to six times on the blossom end with a sterile pipette tip (width: 1 mm, depth: 2 mm) and inoculated with 10 μl per wound using a 500 spores/μl suspension for *B. cinerea*, a 30 spores/μl suspension of *R. stolonifer*, and a 1,000 spores/μl suspension of *F. acuminatum*. The differences in fungal spore concentration were adjusted to ensure uniform and comparable development of lesions in tomato fruit. In the case of *R. stolonifer* inoculations of MG fruit, we also tested a concentration of 1,000 spores/μl but no differences in fruit responses or fungal growth between this concentration and 30 spores/μl were observed. Inoculated fruit were incubated at RT (at approximately 20–23°C) in high humidity (between 90 and 100%) chambers. For mock inoculations, the same procedure was followed but without the addition of the inoculum. The pericarp and epidermis of the blossom end (including both the inoculation point and/or the lesion area) were collected at 1 and 3 days post-inoculation (dpi), immediately frozen in liquid nitrogen, and stored at -80°C until use. One biological replicate consisted on average of eight fruit, and five biological replicates per treatment were obtained.

### RNA Extraction, cDNA Library Preparation, and RNA Sequencing

Tomato fruit tissues were ground using a Retsch^®^ Mixer Mill MM 400 (Retsch, Germany) and RNA was extracted from 1 g of fine-powdered tissue according to the procedure described in Blanco-Ulate et al. (2013). Fungal RNA from the *in vitro* cultures was extracted using TRIzol (Invitrogen, United States) and purified using the Quick-RNA MiniPrep Kit (Zymo Research, United States) following the procedure described in Morales-Cruz et al. (2015). The RNA concentration and purity were assessed with the Qubit 3 (Invitrogen, United States) and the NanoDrop One Spectrophotometer (Thermo Scientific, United States), respectively. Gel electrophoresis was used to confirm the RNA was not degraded. Barcoded cDNA libraries were prepared using the Illumina TruSeq RNA Sample Preparation Kit v2 (Illumina, United States). Quality control of the cDNA libraries was performed with the High Sensitivity DNA Analysis Kit in the Agilent 2100 Bioanalyzer (Agilent Technologies, United States). 50-bp single-end libraries were sequenced on the Illumina HiSeq 4000 platform in the DNA Technologies Core of the UC Davis Genome Center. In total, 18 libraries were sequenced for *B. cinerea* (five for MG 1 dpi, four for RR 1 dpi, and three for MG 3 dpi, RR 3 dpi, and *in vitro* cultures), 17 libraries were sequenced for *F. acuminatum* (four for MG 1 dpi and RR 1 dpi, and three for MG 3 dpi, RR 3 dpi and *in vitro* cultures), and 17 libraries for *R. stolonifer* (five for RR 1 dpi and three for MG 1 dpi, MG 3 dpi, RR 3 dpi, and *in vitro* cultures).

### *De novo* Transcriptome Assembly and Annotation

Quality trimming (*Q* > 30) of the raw reads was performed with sickle v1.33^1^ and adapter sequences were removed with scythe v0.991^2^. The basic quality measurements were assessed with FastQC^3^. To generate transcriptome assemblies for *F. acuminatum* and *R. stolonifer*, reads from samples infected with each of these pathogens were mapped to the tomato genome (ITAG3.2^4^) using STAR 2.6 (Dobin et al., 2013). Reads that failed to map to the tomato gene were pooled with the reads from the *in vitro* samples and used for *de novo* transcriptome assembly. Transcriptomes were assembled with Trinity 2.5.1 (Grabherr et al., 2011) using default parameters (i.e., no specified minimum contig length). Quality of the assemblies was assessed with BUSCO 3.0.2 (Waterhouse et al., 2018) using the “fungi *odb9*” dataset, while basic assembly metrics were obtained with Transrate 1.0.3 (Smith-Unna et al., 2016). Potential contaminant transcripts were identified via BLAST using both the blast nr database and the UniProt database. Transcripts with a top-scoring blast match to *Solanum* species were removed from the final transcriptomes.

Functional annotations for transcriptomes of all three fungi were created using Trinotate 3.0.1 (Bryant et al., 2017). The default Trinotate parameters (-max_target_seqs 1 -outfmt 6 -evalue 1*e*^-3^) were used to retrieve the best BLAST hits as well as annotations for Gene Ontology (GO) terms, Pfam families, Kyoto Encyclopedia of Genes and Genome (KEGG) pathways, EggNOG predictions, and SignalP sequences. Custom BLAST databases were incorporated according to the Trinotate manual for the Transporter Classification Database (TCDB^5^) and the Pathogen-Host Interactions database (PHI^6^). Custom HMMER alignment results for HMM profiles from dbCAN^7^ and fPoxDB^8^ were incorporated similarly.

### RNA-seq Bioinformatics Pipeline

The bioinformatic pipeline was initiated by mapping parsed reads from the fungal-infected tomato samples to a combined transcriptome of tomato and the three pathogens using Bowtie2 (Langmead and Salzberg, 2012). The tomato transcriptome (build SL3.0 with ITAG 3.2 annotations; The Tomato Genome Consortium, 2012) was obtained from https://solgenomics.net/organism/Solanum_lycopersicum/genome. For *B. cinerea* (strain B05.10), we used the predicted transcriptome ASM83294v1 from http://fungi.ensembl.org/Botrytis_cinerea/Info/Index (van Kan et al., 2016). For *R. stolonifer* and *F. acuminatum*, the respective curated transcriptome assemblies were used. The *in vitro* samples were mapped to the pathogen transcriptomes alone. Count matrices were made from the Bowtie2 results using sam2counts.py (v0.91^9^) and are available in Supplementary Table S1.

Count matrices were used as input for differential expression analysis with the Bioconductor package DESeq2 (Love et al., 2014) in R. Reads were normalized for library size after being filtered to exclude those that mapped to tomato. Gene expression comparisons were made between MG or RR and *in vitr*o samples at 1 and 3 dpi. Differentially expressed genes (DEGs) were considered to be those with an adjusted *P*-value less than or equal to 0.05. Enrichment for GO terms was performed with the R package goseq v1.32.0 (Young et al., 2010) with the use_genes_without_cat parameter set to TRUE. An over-represented *P*-value threshold of 0.05 was used to specify enriched genes.

### RT-qPCR Validation

cDNA was synthesized with an oligo-dT primer on 1 μg of RNA using the M-MLV Reverse Transcriptase (Promega, United States) in the SimpliAmp Thermal Cycler (Applied Biosystems, United States). Expression of the genes of interest was quantified through Real-Time Quantitative PCR (RT-qPCR) using PowerSYBR Green PCR Master Mix (Applied Biosystems, United States) in the QuantStudio3 (Applied Biosystems, United States). Primers for the genes of interest were designed using Primer-BLAST (Ye et al., 2012). Primer efficiency was confirmed to be higher than 90% using fourfold DNA or cDNA dilutions (0, 1:1, 1:4, 1:16, 1:64, and 1:256) in triplicate, while specificity was checked by analyzing the melting curves at temperatures ranging from 60 to 95°C. Relative gene expression was calculated using the formula 2^(reference gene Ct – gene of interest Ct)^. The list of primers used in this study are listed in the Supplementary Table S2.

### Data Access

The transcriptome assemblies for *F. acuminatum* and *R. stolonifer* have been deposited at GenBank under the accessions GGXD00000000 and GGWM00000000, respectively. The versions described in this paper are the first versions, GGXD01000000 and GGWM01000000. The RNA-Seq results, including the raw sequencing reads and the read mapping count matrices, have been deposited in the National Center for Biotechnology Information Gene Expression Omnibus (GEO) database^10^ (no. GSE122555).

## Results

### Tomato Fruit Susceptibility to Fungal Infections Increases as a Result of Ripening

**FIGURE 1:**
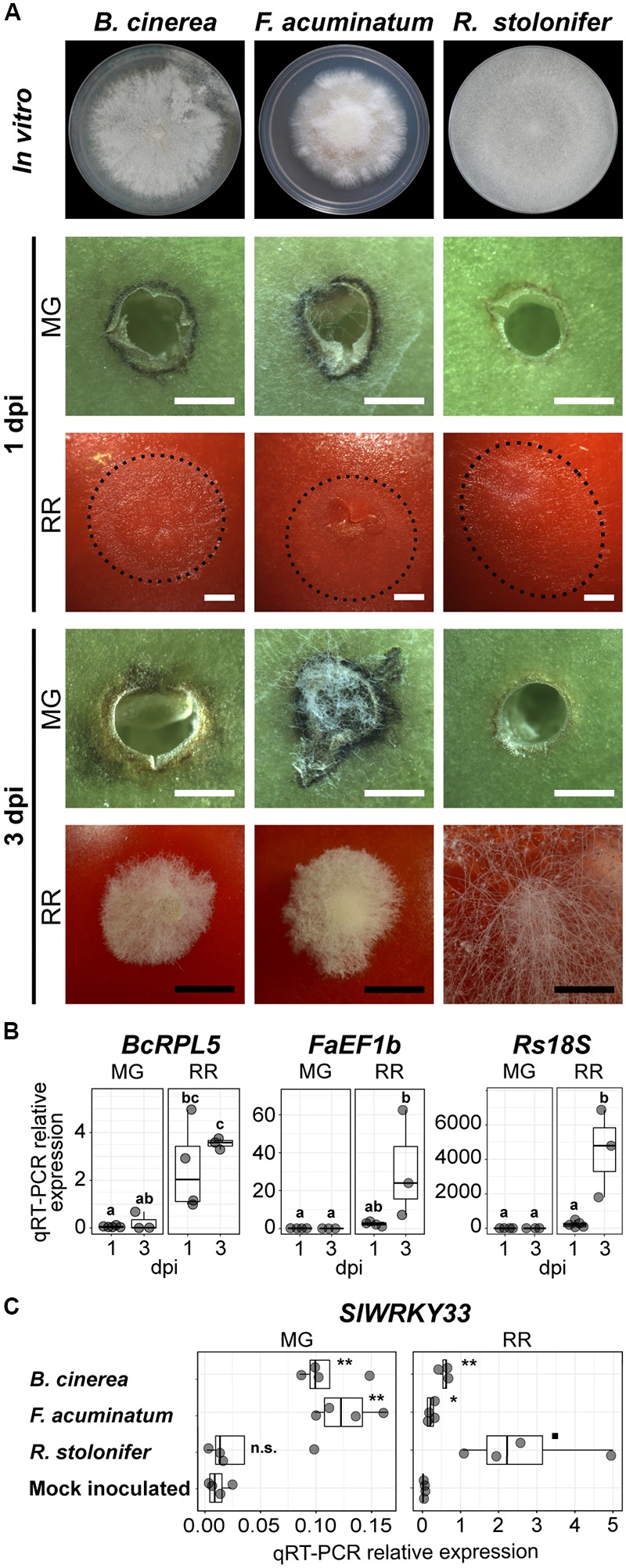
Fungal growth and disease development in tomato fruit. **(A)** Growth and lesion development of the three fungi *in vitro* and during inoculation, respectively. Fungi were grown on PDA in 100 mm Petri dishes. *In vitro* morphology represents the pre-sporulation stage used for this study at 3 (*Rhizopus stolonifer*), 5 (*Botrytis cinerea*) and 7 (*Fusarium acuminatum*) days post-plating. Fungal growth and lesion development during fruit inoculation is shown at 1 and 3 days post-inoculation (dpi) in mature green (MG) and red ripe (RR) fruit. The extent of mycelial growth is highlighted by dotted lines for 1 dpi RR fruit. White and black bars correspond to 1 and 5 mm, respectively. **(B)** Fungal biomass estimated by the relative expression of the reference genes *BcRPL5* (*Bcin01g09620*), *FaEF1b* (*FacuDN4188c0g1i4)*, and *Rs18S* (*RstoDN6002c0g2i1*), normalized based on the tomato reference gene expression (*SlUBQ, Solyc12g04474*). Significant differences (*P* < 0.05) between the biomass of the four treatments are denoted by letters. **(C)** Relative expression of the disease responsive tomato gene *SlWRKY33* (*Solyc09g014990*) in samples inoculated with the three fungi and in the mock-inoculated control. Symbols indicate statistical significance (n.s., not significant; ^◼^*P* < 0.1; ^∗^*P* < 0.05; ^∗∗^*P* < 0.01) when comparing inoculations with each pathogen and the control.

To determine if *F. acuminatum* and *R. stolonifer* show similar patterns of infections in tomato fruit as *B. cinerea* (Cantu et al., 2009; Blanco-Ulate et al., 2013), we did side-by-side inoculations of fruit at two developmental stages: unripe (mature green, MG) and ripe (red ripe, RR). As displayed in Figure 1A, we confirmed that all fungi were unable to cause rotting in MG fruit but aggressively colonized RR fruit. These results were further validated by quantifying fungal biomass based on relative expression of fungal reference genes via qRT-PCR (Figure 1B). At 3 dpi, RR fruit inoculated with *B. cinerea* and *F. acuminatum* showed water-soaked lesions of approximately 15 mm covered by dense mycelia, whereas RR fruit inoculated with *R. stolonifer* were almost decomposed and entirely covered by mycelia. Although no lesions were observed in MG fruit when inoculated with any of the pathogens, some differences in fungal growth and tomato responses were observed. Inoculations with *B. cinerea* and *R. stolonifer* did not show any visible mycelia, whereas *F. acuminatum* inoculations showed limited hyphal growth without disease symptoms. All three fungi induced a necrotic ring surrounding the inoculation sites during the incompatible interaction with MG fruit, yet *F. acuminatum* inoculations caused dark and wide rings while fruit infected with *R. stolonifer* developed a weaker response. Because we were not able to visually detect any hyphal growth of *B. cinerea* and *R. stolonifer* in MG fruit, we used a microscope to observe whether the spores germinated in the inoculated wounds. At 1 dpi, *B. cinerea* spores were mainly ungerminated or in the process of germination (Supplementary Figure S1). By contrast, *F. acuminatum* and *R. stolonifer* already showed active hyphal growth, indicating that spores of these fungi germinate earlier on MG fruit. At 3 dpi, some hyphal growth was also observed for *B. cinerea*. Together, these observations suggest that the incompatibility of the interaction between these fungi and MG tomato fruit occurs after spore germination.

To provide initial support that both *F. acuminatum* and *R. stolonifer* are capable of inducing disease responses in the host, like *B. cinerea*, and do not merely behave as saprotrophs (i.e., feeding on dead tissue), we evaluated the expression of the host gene *SlWRKY33* (*Solyc09g014990*), which is well-known to be pathogen-responsive but is not induced by abiotic stresses (Zhou et al., 2014, 2015). To test that the induction of this gene occurred only as a result of inoculation and not wounding, we included a mock-inoculated control in our analyses. The expression patterns of *SlWRKY33* measured by qRT-PCR reflected the accumulation of fungal biomass and the presence of lesions in each of the treatments (Figure 1C). At 1 dpi, expression of *SlWRKY33* was induced by inoculation with both *B. cinerea* and *F. acuminatum* but not with *R. stolonifer* or mock inoculation in MG fruit. In RR fruit, pathogen-induced *SlWRKY33* was detected for all three pathogens at greater levels than found in MG fruit.

### Novel Transcriptomic Resources for *F. acuminatum* and *R. stolonifer*

Our observations of lesion development, fungal biomass, and activation of pathogen responses led to the hypothesis that *F. acuminatum* and *R. stolonifer* display a similar necrotrophic behavior in tomato fruit as *B. cinerea*. Therefore, to discover pathogenicity or virulence factors in these fungi that are important for necrotrophic infections, we performed a genome-wide transcriptomic analysis of inoculated fruit at both time points as well as *in vitro* cultures. Due to the lack of publicly available genomic data for *F. acuminatum* and *R. stolonifer*, we assembled *de novo* transcriptomes for both of these pathogens from our cDNA libraries following the Trinity pipeline (Grabherr et al., 2011; see Section “Materials and Methods” for details). Using the fungal ortholog dataset of the Benchmarking Universal Single-Copy Orthologs tool (BUSCO v3; Waterhouse et al., 2018), we determined that our assemblies presented high completeness, with 88.2 and 90.3% of *F. acuminatum* and *R. stolonifer* matches being complete, respectively. Our *F. acuminatum* transcriptome contained 20,117 unique transcripts, while our *R. stolonifer* transcriptome contained 19,754 (see Table 1).

**Table 1:** Quantitative summary of *de novo* assembled transcriptomes of *F. acuminatum* and *R. stolonifer*.

	*F. acuminatum*	*R. stolonifer*
Transcripts in initial assembly	20,446	20,099
Removed contaminant transcripts	329	345
Transcripts in final assembly	20,117	19,754
Transcripts with ORF	9,617	11,468
N50 (bp)	1,825	1,412
Transcripts annotated	10,432	13,049


*ORF, open reading frame. N50 refers to the minimum transcript length required to cover 50% of the transcriptome.*

**FIGURE 2:**
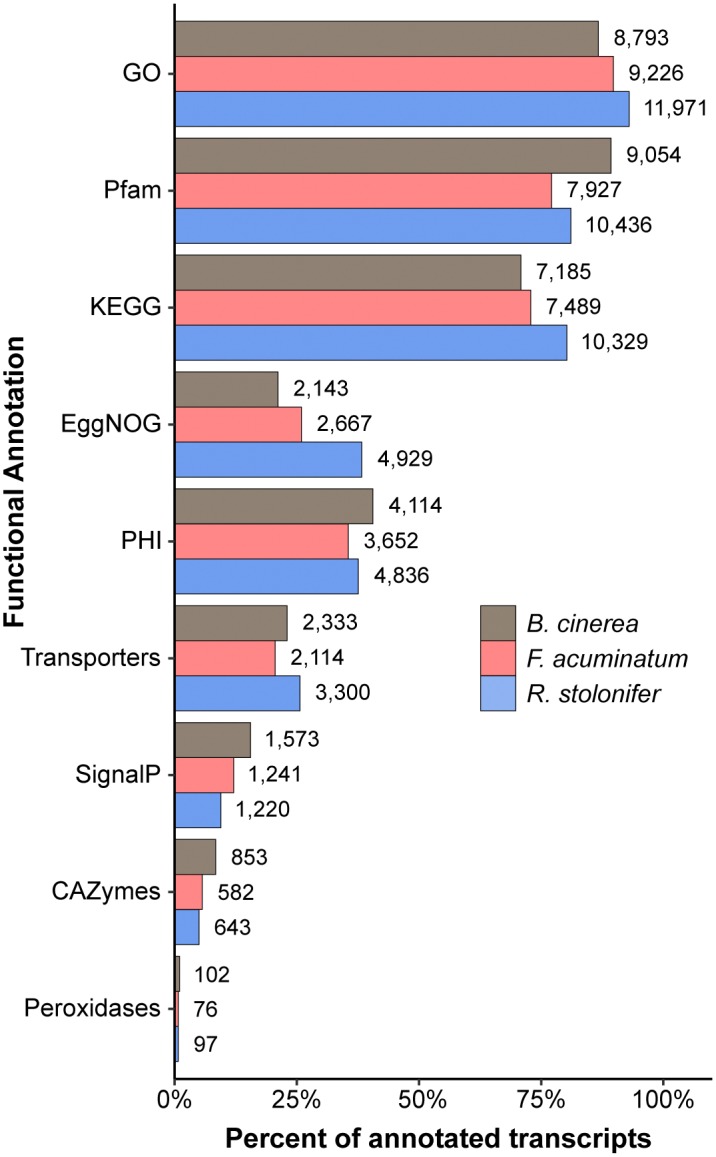
Summary of functional annotations across the *Botrytis cinerea*, *Fusarium acuminatum*, and *Rhizopus stolonifer* transcriptomes. Percent of all annotated transcripts in that transcriptome that contain at least one annotation for each categorization. GO, Gene Ontology; KEGG, Kyoto Encyclopedia of Genes and Genomes; PHI, Pathogen-Host Interaction; CAZymes, Carbohydrate-Active enZymes. Detailed information can be found in Supplementary Table S3.

We then used homology-based annotation to obtain information on gene functions for each of the transcriptomes, including the *B. cinerea* B05.10 ASM83294v1 (van Kan et al., 2016). We annotated transcripts based on nine separate functional classifications, including GO (The Gene Ontology Consortium, 2017), Pfam domains (El-Gebali et al., 2018), Pathogen-Host Interaction (PHI; Urban et al., 2017), membrane transporters (Saier et al., 2016), Carbohydrate-Active Enzymes (CAZymes; Lombard et al., 2014), and fungal peroxidases (Choi et al., 2014). Each type of functional annotation was represented by a similar percentage of annotated transcripts across all pathogens (Figure 2). The specialized enzyme classifications of peroxidases and CAZymes made up a relatively small fraction of the annotated transcripts, whereas general functional classifications such as GO, Pfam, and KEGG descriptions were available for at least 70% of the annotated transcripts for all pathogens. Annotations for all three transcriptomes can be found in Supplementary Table S3. Although the *F. acuminatum* and *R. stolonifer* transcriptomes are preliminary and may require further curation and validation, we consider that they are a valuable resource to perform gene expression analyses and to shed light on the infection strategies utilized by these fungi.

### Fungal Gene Expression Patterns Are Distinct During Interactions With Unripe and Ripe Fruit

**FIGURE 3:**
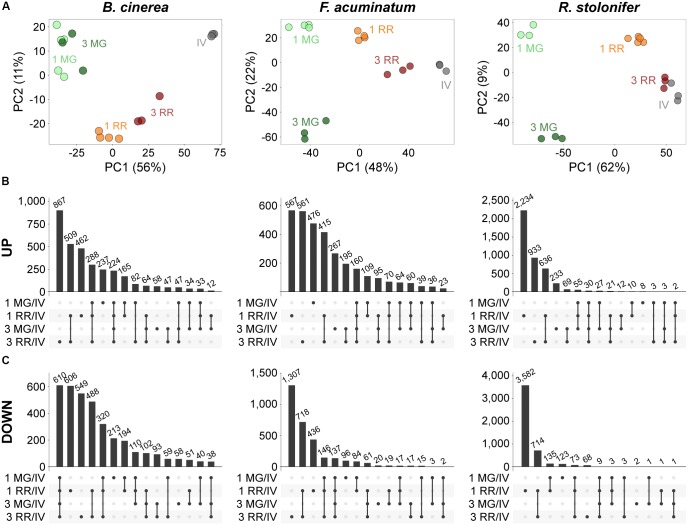
Principal component analysis (PCA) and intersections of differentially expressed genes (DEGs) during inoculations of tomato fruit. **(A)** PCA plots of variance-stabilized matrixes of mapped reads for each pathogen as generated by DESeq2. **(B,C)** UpSetR visualizations of intersections between the upregulated **(B)** and downregulated **(C)** DEGs of inoculated fruit at two time points versus *in vitro* comparisons for each pathogen. Intersections are displayed in descending order by number of genes. All datasets can be accessed in Supplementary Tables S5–S7.

First, we performed principal component analysis (PCA) to determine if the fungal-inoculated and *in vitro* samples could be discerned based on the expression of the fungal transcripts. The PCAs revealed that all samples clustered by treatment (Figure 3A). In most cases, the first component clearly differentiated the MG fruit from the RR fruit inoculations and the *in vitro* samples. Then, we determined DEGs (*P*_adj_ ≤ 0.05) between inoculations of MG or RR fruit and *in vitro* cultures for each pathogen. Across all comparisons, we detected 6,488 *B. cinerea* DEGs (47.19% of its transcriptome), 6,154 *F. acuminatum* DEGs (30.59% of its transcriptome), and 8,777 *R. stolonifer* DEGs (44.43% of its transcriptome). The number of DEGs for *R. stolonifer* were mainly identified in the RR fruit comparisons, as the low amount of fungal biomass in MG fruit samples did not allow for an in-depth sequencing coverage of the fungal transcripts. To confirm the accuracy of the DEG analysis, we selected a subset of genes for each pathogen to validate their expression using a qRT-PCR approach (Supplementary Table S4). Our results confirmed that the gene expression values were consistent, showing significant Pearson correlation coefficients (*r* ≥ 0.7, *P* < 0.05) and between the RNA-seq and the qPCR expression data (Supplementary Figure S2).

We further evaluated the fungal DEGs based on whether they were commonly or uniquely expressed under specific treatments, which can provide insight on particular sets of genes that are relevant during incompatible or compatible interactions (Supplementary Tables S5–S7). For each pathogen, genes uniquely upregulated in RR fruit (Figure 3B) constituted a sizable fraction of upregulated genes (58.80%, 49.19%, and 88.94% for *B. cinerea*, *F. acuminatum*, and *R. stolonifer*, respectively). This result may be influenced by the fact that RR fruit samples had more coverage of fungal transcripts in the RNA-seq experiment than MG fruit samples, which is a technical limitation of this type of study. Nevertheless, the comparisons of common and unique DEGs among treatments for each of the pathogens support the results of the PCAs, indicating that these fungi display a specific behavior in each of the fruit stages at early and late time points after inoculation. We also identified upregulated DEGs shared across categories (224 for *B. cinerea*, 160 for *F. acuminatum*, and 30 for *R. stolonifer*) that are likely to represent core pathogenicity factors during fruit infections.

### Necrotrophic Fungi Utilize Similar, Yet Distinct Infection Strategies in Tomato Fruit

**FIGURE 4:**
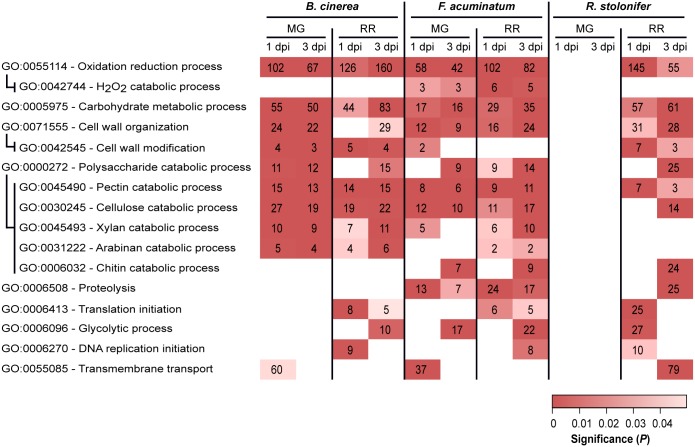
Subset of overrepresented Gene Ontology (GO) terms associated with pathogenicity and fungal growth among upregulated genes in *Botrytis cinerea*, *Fusarium acuminatum* and *Rhizopus stolonifer* inoculated samples. Indented GO terms are nested in the GO term above. Box color indicates the significance of enrichment, and values in the boxes indicate the number of upregulated genes in this comparison that share the indicated annotation. dpi, days post-inoculation; MG, mature green; RR, red ripe. Supplementary Table S8 includes the complete list of overrepresented GO terms.

To gain insight into key biological processes that are relevant during compatible or incompatible fruit infections, we performed GO enrichment analyses of the upregulated DEGs in all combinations of ripening stage (MG and RR) and dpi (1 and 3) for each pathogen (Supplementary Table S8). We mainly focused on GO terms of the “biological process” class that were significantly enriched (*P* < 0.05, number of genes ≥ 2) and appeared to be involved in pathogenesis or fungal growth in the host tissues (Figure 4).

Upregulated DEGs from all comparisons, except for *R. stolonifer* MG inoculations, were enriched in oxidation-reduction processes (GO: 0055114). A closer inspection of these DEGs revealed functions that are likely to be involved with pathogenicity, such as catabolism of ROS [e.g., superoxide dismutases (SODs), catalases (CATs), peroxidases] and breakdown of cell wall molecules such as cellobiose and lignin (Supplementary Table S8). In *B. cinerea*, the SOD *BcSOD1* was induced in both MG and RR fruit at 1 and 3 dpi. Additionally, *BcSOD3* (*Bcin01g03830*) was upregulated only in MG fruit at 1 dpi, and *BcSOD2* (*Bcin01g03830*) is upregulated only at 1 dpi in MG and RR fruit. Although two potential SODs, *FacuDN9613c0g1i1* and *FacuDN4275c0g1i2*, were employed by *F. acuminatum* in all treatments except 1 dpi MG, none of the seven putative SODs identified in *R. stolonifer* were upregulated in any of the treatments. To further identify enzymatic scavengers of hydrogen peroxide (H_2_O_2_), we examined the upregulated DEGs of each pathogen which showed significant (*E* ≤ 1*e*^-3^) similarity to members of the Fungal Peroxidase Database. This analysis revealed differences both in the classes of enzymes used in each pathogen and the treatments in which they were used. For example, in *B. cinerea*, only two known catalases, *BcCAT2* (*Bcin11g06450*) and *BcCAT4* (*Bcin05g00730*), were found to be upregulated during tomato fruit interaction. Both of these were only active in MG fruit. In contrast, *F. acuminatum* exhibited very strong induction (log_2_FC > 9) of two predicted CATs, *FacuDN12367c0g1i1* and *FacuDN13048c0g1i1*, at 1 dpi in RR fruit but not in MG fruit, although a handful of CATs and catalase-peroxidases were upregulated less strongly across both MG and RR fruit. In all *F. acuminatum*-inoculated samples, there was also an enrichment of DEGs involved in hydrogen peroxide catabolism (GO: 0042744), further highlighting the importance of fungal responses to oxidative stress during fruit colonization. In *R. stolonifer*, peroxidases were only upregulated at 1 dpi in RR fruit and included two 2-cysteine peroxiredoxins (PRXs), one cytochrome C peroxidase, and one glutathione peroxidase (GPX).

Additionally, in all *B. cinerea*-inoculated samples, DEGs annotated with the oxidation-reduction process GO term included enzymes in the biosynthetic pathways for the phytotoxins botrydial and botcinic acid. Eight of these genes were strongly upregulated (log_2_FC > 4) in all four treatments, indicating that *B. cinerea* may produce these toxins regardless of the ripening stage of the fruit. *F. acuminatum* genes annotated with this GO term included enzymes involved in the biosynthesis of the toxin fumonisin. Several of these genes (*FacuDN12063c0g1i1*, *FacuDN15813c0g1i1*, *FacuDN9039c0g1i1*) showed significant upregulation (log_2_FC > 2) in infections of MG fruit at 1 dpi or RR fruit at both time points. Fungal proteolysis-related genes (GO: 0006508) were found to be enriched during MG and RR inoculations with *F. acuminatum* as well as RR inoculations with *R. stolonifer* at 3 dpi. Though not enriched, several genes with this GO term were also found to be expressed during fruit inoculation by *B. cinerea*, mostly in RR fruit. Across all treatments, *F. acuminatum* was found to produce 28 genes with this GO term, while *B. cinerea* was found to produce 29, and *R. stolonifer* produced 44 in RR fruit alone (Supplementary Table S8). Seven members of the *B. cinerea* aspartic proteinase family (ten Have et al., 2010) were upregulated in at least one of the fruit inoculations, though none were upregulated at 1 dpi in RR fruit. Thus, fungal proteases are likely to be a strategy used by all three pathogens.

Other GO terms served as a proxy for successful growth. Enrichments of genes involved in protein translation initiation (GO: 0006413), glycolytic process (GO: 0006096), and DNA replication initiation (GO: 0006270) were found in compatible interactions with RR fruit. Notably, DEGs involved in glycolytic process were enriched in MG inoculations for *F. acuminatum* at 1 dpi, which is consistent with visual observations of mycelium growth on inoculated fruit. A similar pattern was observed for the chitin catabolic process (GO: 0006032) term, which are involved in the continuous fungal cell wall remodeling during hyphal growth (Langner and Göhre, 2016).

**FIGURE 5:**
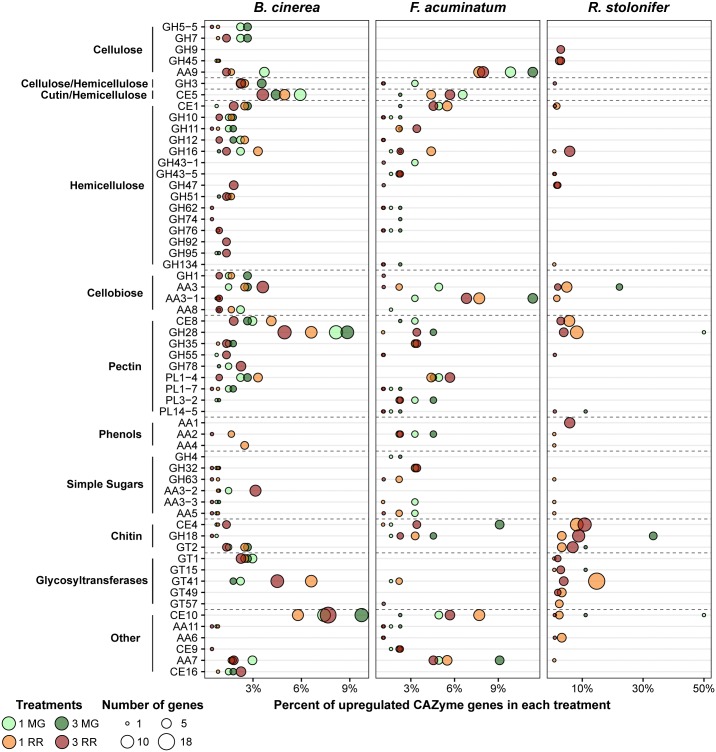
Upregulated CAZy family genes for each pathogen in each of the four treatments. Families and subfamilies from CAZy (www.cazy.org) are listed on the left. These are further nested into categories based on their substrates or activities. Each family is described by the percentage of all upregulated CAZyme genes it represents in each treatment. Only families which constitute at least 2% of upregulated CAZyme genes in at least one treatment are shown. All remaining CAZy families can be retrieved from Supplementary Table S9.

Multiple GO terms relating to carbohydrate metabolism were found to be enriched across multiple fruit inoculation treatments. The corresponding genes included those involved in breakdown of the cell wall polysaccharides, metabolism of host sugar sources, and production of fungal polysaccharides. As both the cell wall properties and sugar biochemistry differ between MG and RR fruit, we hypothesized that the fungi employ different classes of CAZymes depending on the ripening stage as already demonstrated for *B. cinerea* (Blanco-Ulate et al., 2014). To test this, we examined the expression profiles of CAZyme families among the DEGs for each pathogen (Figure 5 and Supplementary Table S9). CAZyme families involved in catabolism of cellulose, hemicellulose, pectin, and monosaccharides were detected, along with families with non-carbohydrate substrates and several responsible for polysaccharide biosynthesis. In *B. cinerea* and *F. acuminatum* inoculations, families involved in the degradation of cellulose (GH5, GH7, AA9) and hemicellulose (AA9 and multiple GHs) were more prominent during infections of MG fruit than RR fruit. Moreover, the CE5 family, which contains cutinases and acetylxylan esterases, was also especially utilized at 1 dpi in MG fruit. In *B. cinerea*, this family included the *cutA* gene previously shown to be expressed in tomato fruit infection (van Kan et al., 1997). MG infections also exhibited higher percentages of families involved in the degradation of cellobiose, a disaccharide of β-1,4-linked glucose molecules that results from the breakdown of cellulose and glucan-based hemicelluloses. A similar trend was found for pectin-degrading families, particularly polygalacturonases (GH28) and pectate lyases (PL1 and PL3), though the PL1-4 subfamily appeared to be prominent in RR infections as well. Enzymes involved in metabolism of simple sugars, most notably GH32 in *F. acuminatum* and AA3-2 in *B. cinerea*, showed greater prominence in RR infections. Chitin and chitosan biosynthesis and processing families (GT2, GH18, CE4) were also detected in *B. cinerea* and *F. acuminatum*. In *B. cinerea*, chitin synthases (GT2) were generally equally expressed in all fruit inoculations, though chitin deacetylases (CE4), which produce chitosan, were only particularly prominent in RR infections at 3 dpi. In contrast, *F. acuminatum* produces multiple CE4 enzymes at 3 dpi in MG infections in addition to RR infections.

Other CAZy families also seemed to be featured heavily in fruit-pathogen interactions. CE10 enzymes were especially prevalent in both *B. cinerea* and *F. acuminatum* infections. Members of the CE10 family include lipases, which catalyze the hydrolysis of fatty acids. The previously described *B. cinerea* gene *lip1* (Reis et al., 2005) was upregulated at both 1 and 3 dpi in MG fruit, but not RR fruit. Additionally, *B. cinerea* and *F. acuminatum* both produced multiple AA7 family enzymes in both MG and RR fruit. Many of these genes showed significant similarity to three genes of the PHI database: *ZEB1* in *F. graminearum*, *CTB5* from *Cercospora nicotianae*, and *sol5* from *Alternaria solani*. Each of these PHI genes is involved in the biosynthesis of polyketide mycotoxins in those plant pathogens (Chen et al., 2007; Kim et al., 2015; Park et al., 2015). Thus, these *B. cinerea* and *F. acuminatum* genes may be involved in similar roles.

Detection of CAZymes during infection by *R. stolonifer* was only possible in RR fruit due to the low number of DEGs determined in MG fruit. However, sizable numbers of genes from families detected in *B. cinerea* and *F. acuminatum* infections were also discovered in *R. stolonifer*. These include xyloglucanases (GH16), cellobiose dehydrogenases (AA3), pectin methylesterases (CE8), and polygalacturonases (GH28). In addition, multiple enzymes involved in chitin/chitosan biosynthesis were prevalent in RR fruit inoculations, which is indicative of the particularly aggressive hyphal growth of *R. stolonifer* on these fruit. In RR fruit at 3 dpi, *R. stolonifer* also produced six enzymes of the AA1 family, which consist of laccases, ferroxidases, and multi-copper oxidases. Each of these enzymes showed significant similarity to *FET3* enzymes from *Colletotrichum graminicola* in the PHI database and to genes of the TCDB class 2.A.108.1.4, the latter being iron transport multicopper oxidase *FET5* precursors. This finding is also consistent with the enrichment of transmembrane transport genes (GO: 0055085) during RR infection at 3 dpi for *R. stolonifer*.

### Infections of Non-ripening Tomato Fruit Are Comparable to Infections of Unripe Tomato Fruit

**FIGURE 6:**
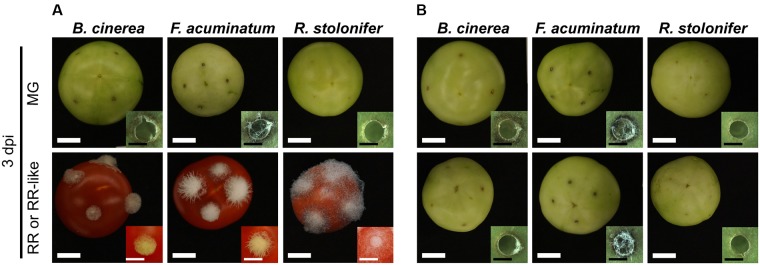
Fungal pathogens are unable to infect fruit from the *non-ripening* (*nor*) tomato mutant. **(A)** Shows inoculations of *Botrytis cinerea*, *Fusarium acuminatum* and *Rhizopus stolonifer* on mature green (MG) and red ripe (RR) wild-type tomato (cv. Ailsa Craig) fruit at 3 days post-inoculation (dpi). **(B)** Shows inoculations of the same fungi on MG and RR-like fruit from the isogenic non-ripening (*nor*) tomato mutant. White size bars correspond to 1 cm whereas black size bars correspond to 1 mm.

We inoculated fruit of the *non-ripening* (*nor*) tomato mutant to verify the effect of the ripening stage on the infection success of *B. cinerea*, *F. acuminatum* and *R. stolonifer*. Fruit from the *nor* mutant do not show ripening-associated processes, such as carotenoid and sugar accumulation or cell wall disassembly, and therefore resemble wild-type MG fruit even at a comparative RR-like stage. None of the three pathogens were able to infect *nor* fruit at any ripening stage (Figure 6 and Supplementary Figure S3). No hyphal growth of *B. cinerea* and *R. stolonifer* was apparent, whereas *F. acuminatum* formed visible mycelia especially at 3 dpi in MG and RR-like fruit. Like on wild-type MG fruit, all three fungi induced necrotic rings in *nor* fruit. When inoculated in RR-like fruit from *nor*, the three fungi displayed similar growth and morphology as in MG fruit from wild-type and *nor*, indicating that for compatible interactions to occur, tomato fruit needs to undergo certain ripening processes that facilitate fungal colonization and spread.

## Discussion

Successful infections of *B. cinerea*, *F. acuminatum* and *R. stolonifer* in tomato fruit are dependent on the host developmental stage. In MG fruit, all three fungi were able to grow on the surface, but none of the pathogens was able to cause rot. In contrast, ripe tomato fruit represented a compatible system for infections as all three fungi induced lesions that spread rapidly. This contrasting ability to cause disease in fruit has been previously reported for a variety of fungal pathogens, particularly those displaying necrotrophic behavior (Prusky and Lichter, 2007; Cantu et al., 2008; Guidarelli et al., 2011; Blanco-Ulate et al., 2016b). The tomato gene *SlWRKY33* has been shown in leaves to be expressed in response to *B. cinerea* inoculation, and deletion of this gene leads to increased susceptibility, indicating its role in defense response (Zhou et al., 2015). We demonstrated that, as in leaves, *B. cinerea* is capable of inducing *SlWRKY33* in MG and RR fruit. Furthermore, *F. acuminatum* also induced *SlWRKY33* in MG and RR fruit, and *R. stolonifer* did so substantially in RR fruit. These findings indicate that all three pathogens triggered disease responses in the host and that the strength of the response was reflective on the success of the infection process.

During interactions with tomato fruit, *B. cinerea*, *F. acuminatum*, and *R. stolonifer* employed a variety of pathogenicity and survival strategies that involved redox processes, carbohydrate catabolism, and proteolysis. Moreover, the degree to which particular strategies were used varied according to the ripening stage of the fruit, as certain processes were emphasized in either MG (such as pectin, cellulose, cellobiose. and hemicellulose breakdown by *B. cinerea* and *F. acuminatum*) or RR fruit (e.g., growth-related processes such as translation initiation, and DNA replication initiation and simple sugar catabolism). These observations suggest that the fungi can sense the physiological environment of the fruit and react accordingly with suitable infection, growth, or quiescence strategies. Though these fungi are incapable of causing disease symptoms in MG tomato fruit, this study demonstrates that they do make attempts to either establish infections or create a suitable environment in fruit for fungal growth and do not merely die on the host tissues. However, when the conditions in fruit are highly unsuitable (e.g., strong cell walls, prevalent antifungal compounds, active host defenses), the infection strategy of the fungal pathogen is often insufficient to cause successful infections. In many cases, when fungal pathogens encounter incompatible conditions, like in unripe fruit, they enter a quiescent phase with limited growth and activity (Prusky et al., 2013). During ripening, the physicochemical properties of the fruit tissues change, resulting in compatible conditions for the fruit-pathogen interaction and the reactivation of quiescent pathogens (Prusky and Lichter, 2007). In this context, it would also be interesting to investigate the strategies employed by the three fungi during inoculations of other plant organs such as leaves. Our initial tests, however, indicated that both *F. acuminatum* and *R. stolonifer* are incapable of infecting tomato leaves even when leaves were senescing. This observation may suggest that the isolates of these two fungi are exclusive fruit pathogens and lack the molecular toolset to grow on leaves.

The redox environment of the plant-pathogen interface influences the outcome of the interaction. Upon pathogen detection, ROS are rapidly produced by the host, triggering a downstream signaling of various defense responses (Camejo et al., 2016). The enzymatic agents of this oxidative burst are respiratory burst oxidative homologs (RBOHs), which generate superoxide $O_{2}^{–}$ in the apoplast (Suzuki et al., 2011). This oxidative burst has been previously reported in incompatible tomato-*Botrytis* interactions (Asselbergh et al., 2007), including MG fruit, in which the appearance of a necrotic ring is associated with resistance to *B. cinerea* (Cantu et al., 2009; Blanco-Ulate et al., 2013). However, necrotrophic pathogens can exploit this ROS response by overwhelming the host with their own ROS production (Siegmund and Viefhues, 2015). In leaves of French bean (*Phaseolus vulgaris*), *B. cinerea* has been shown to produce ROS as virulence factors by activating the NADPH oxidases *BcnoxA* (*Bcin05g00350*) and *BcnoxB* (*Bcin02g04930*), coupled with the regulatory protein *BcnoxR* (Bcin03g06840; Siegmund et al., 2013). Although we did not detect strong upregulation of these genes during inoculation of fruit, other ROS producing systems, including laccases (e.g., *Bclcc8*, *Bcin01g00800*) and glucose oxidases (e.g., *BcGOD1*, *Bcin14g05500*) were upregulated during inoculations of tomato fruit. In *F. acuminatum*, a *BcnoxA* homolog *FacuDN4838c0g1i1* and *BcnoxB* homolog *FacuDN3221c0g1i1* were induced in specific treatments. A BLAST search did not reveal any homologs of *BcnoxA* or *BcnoxB* in *R. stolonifer*, nor were any homologs of *Bclcc8* or *BcGOD1* detected in either *F. acuminatum* or *R. stolonifer*.

In addition to ROS generation machinery, fungal pathogens must protect themselves against the oxidative stress of the infection site. Methods of ROS scavenging in phytopathogenic fungi include enzymatic and non-enzymatic mechanisms (Heller and Tudzynski, 2011). SODs catalyze the conversion of O_2_^-^ produced by RBOHs into the less reactive hydrogen peroxide (H_2_O_2_). *B. cinerea* mutants lacking the *BcSOD1* (*Bcin03g03390*) gene have been shown to have reduced virulence on tomato leaves (López-Cruz et al., 2017). In tomato fruit, *BcSOD1* is upregulated for both MG and RR ripening stages, which suggests it is also a critical gene for fruit colonization. H_2_O_2_ can be converted to water by either catalases (CATs) or peroxidases such as GPXs or PRXs. All three pathogens demonstrated upregulation of specific mechanisms of catabolizing H_2_O_2_, but only *F. acuminatum* showed enrichment of genes involved in the H_2_O_2_ catabolic process. The usage of these H_2_O_2_ catabolizing systems varied between the pathogens. While *B. cinerea* utilized catalases in MG fruit at 1 dpi, *F. acuminatum* and *R. stolonifer* produced more catalases and peroxidases in RR fruit at 1 dpi.

In each pathogen, multiple genes involved in protein degradation were found to be upregulated during fruit inoculations. The strong enrichment of proteolysis-related genes may indicate that protein degradation is important for pathogenicity of *F. acuminatum* and *R. stolonifer* but not *B. cinerea*. Some pathogen-derived proteases, such as Sep1 and Mep1 in *Fusarium oxysporum*, are known to serve as suppressors of host-immune response in plant-pathogen interactions (Hou et al., 2018). Even though their specific roles in pathogenesis are not fully characterized, several aspartic proteinases in *B. cinerea* have been described (ten Have et al., 2010). Three of the *B. cinerea* aspartic proteinases (*BcAP5*, *BcAP8*, and *BcAP9*) that we found to be induced in tomato fruit were also found to be upregulated during infection of grape berries (Kelloniemi et al., 2015). Aspartic proteinases were also found to be among the upregulated proteinases in *F. acuminatum* (4 genes) and *R. stolonifer* (31 genes), though all three pathogens appeared to utilize a diverse suite of proteinases of different families. Especially prominent in *F. acuminatum* and *R. stolonifer* were proteins with similarity to subtilisin-like proteases. This family of enzymes is mostly associated with plants and particularly plant defense, but subtilisin-like proteases involved in pathogenicity have been described for fungi as well (Figueiredo et al., 2018). Fungal plant pathogens are also known to express inhibitors of these types of proteases as a counter-defense (Figueiredo et al., 2018). Since these inhibitors possess sequence similarity to the proteases themselves, the enzymes identified in *F. acuminatum* and *R. stolonifer* may be inhibitors, proteases, or a mixture of both. Additionally, proteases can help with host tissue decomposition by breaking down cell wall structural proteins or can serve in degradation of proteins to provide a source of nutrition for fungal growth (Lebeda et al., 2001). For example, the saprotrophic fungal species *Verticillium albo-atrum* and *V. dahliae* were described to secrete proteases to break down structural proteins that stabilize the plant cell walls (Leger et al., 1997). High proteolytic activity resulting in the degradation of proteins into free amino acids was also reported during fermentation of tempeh by several *Rhizopus* species (Baumann and Bisping, 1995).

*Botrytis cinerea*, *F. acuminatum*, and *R. stolonifer* also make use of a variety of CAZymes during interactions with the host. Several CAZyme families are involved in the breakdown of physical barriers present in the host tissues, namely the various cell wall components (cellulose, hemicellulose, and pectin), cell wall reinforcements (lignin), and the waxy fruit cuticle. Many of these enzymes, such as polygalacturonases, pectin methylesterases, pectate lyases, and endo-β-1,4-glucanases, mirror the activities of host enzymes active during the ripening-related softening of the fruit (Bennett and Labavitch, 2008). Others, such as cellulases, cutinases, and lipases, degrade components that are not typically degraded during ripening. Production of cellulases is also coupled with enzymes involved in degradation of cellobiose, the disaccharide product of cellulose breakdown. Both *B. cinerea* and *F. acuminatum* appear to focus on production of these latter CAZyme families (e.g., GH5-5, GH7, AA9, CE5, CE10) in MG fruit more than in RR fruit. This may be due to the greater strength and integrity of the cell wall in MG fruit, which requires the fungus to mount a larger attack on the physical barriers in order to penetrate into the cells.

Degradation of pectin is a hallmark feature of *B. cinerea* infection of plant tissues (Choquer et al., 2007; Lionetti et al., 2007; Shah et al., 2009; Blanco-Ulate et al., 2014). The principal enzymes responsible for this process are polygalacturonases (PGs, GH28), pectin methylesterases (PMEs, CE8), and pectate lyases (PLs, PL1, PL3). Both PGs and PLs cleave the α-1,4-linkages in the homogalacturonan backbone of pectins. PMEs catalyze the removal of methylester groups on the C6 carbons of galacturonan, which allows for further degradation by PGs. Although overexpression of PME inhibitors in Arabidopsis leaves has been shown to increase resistance to *B. cinerea* (Lionetti et al., 2007), mutations in *Bcpme1* and *Bcpme2* do not appear to affect virulence in tomato leaves (Kars et al., 2005). In *B. cinerea*, all three classes of enzymes appear to be highly expressed in MG fruit but not as prominently in RR fruit. Not only do the GH28, PL1-7, and PL3-2 families constitute a greater fraction of upregulated CAZymes in MG fruit, but for PGs, PLs, and PMEs that are commonly upregulated in MG and RR fruit, upregulation is consistently greater (by differential expression analysis) in MG fruit over RR fruit. Additionally, although no *F. acuminatum* PGs were detected in MG, the two upregulated PMEs, *FacuDN5818c0g1i1* and *FacuDN10179c0g1i1*, were only active in MG fruit. Moreover, PL1-7 and PL3-2 genes were strongly expressed in MG fruit, with one PL3-2 gene, *FacuDN8473c0g1i1*, showing a log_2_FC of 10.29 at 1 dpi, the highest of any plant CWDE in this treatment. Only one *R. stolonifer* PG, *RstoDN2036c0g1i1*, was detected in MG fruit. However, given that this single *R. stolonifer* PG was one of only two CAZymes found in 1 dpi MG fruit, it is reasonable to believe PG activity in *R. stolonifer* is being underestimated due to low sequence coverage of fungal transcripts in this treatment. The absence of upregulation of any *R. stolonifer* pectate lyases in any fruit further underscores this point. Given the prominence of pectin degradation in *B. cinerea* and *F. acuminatum*, a more targeted analysis of *R. stolonifer* pectin degradation, especially in MG fruit, is warranted.

Degradation of the host cell wall in MG fruit by pathogen enzymes may accelerate ripening and in turn facilitate a more favorable environment for colonization. Pectin-derived oligosaccharides have been shown to induce ethylene production in tomato fruit (Bennett and Labavitch, 2008), which further upregulates expression of host CWDEs, including PG. *B. cinerea* can synthesize its own ethylene via the α-keto-γ-methylthiobutyric acid (KMBA) pathway (Cristescu et al., 2002), though it is still unknown whether the pathogen produces ethylene during interactions with the fruit. Ethylene production during plant infection has also been reported via the KMBA pathway for species of *Fusarium* (Tzeng and DeVay, 1984; Ansari et al., 2013), but not, to our knowledge, for *R. stolonifer*. However, the specific genes involved in the KMBA pathway in *B. cinerea* or *Fusarium* spp. have yet to be elucidated.

As colonization proceeds, sugar substrates become available due to degradation of cell wall polysaccharides as well as increased access to stored sugars in the fruit. As a consequence, fungi actively infecting RR tomato fruit induced enzymes (GH32, AA3-2) that metabolize simple sugars. Sugar metabolism is accompanied by expression of CAZyme families involved in the production and modification of chitin, the structural component of fungal cell walls. Chitin production is known to be a hallmark of growth for fungal pathogens (Lenardon et al., 2010). Interestingly, chitin production and modification appear to be prominent not only in RR fruit for each pathogen, but also in MG fruit inoculated with *F. acuminatum*, where a much greater amount of mycelia growth was observed compared to the other two pathogens. The equal representation of CE4 enzymes in MG and RR fruit inoculated with *F. acuminatum* is reflective of the ability of this fungus of producing hyphae at either fruit ripening stage. The abundance of polysaccharide-building glycosyltransferases in RR infections with *R. stolonifer* is also likely connected to the abundant mycelial growth.

Other CAZyme families represent more specialized roles in the infection process. Production of enzymes in the AA7 family may be related to the production of polyketide toxins in *B. cinerea* and *R. stolonifer*. *B. cinerea* is known to produce botcinic acid, a polyketide mycotoxin, during infection (Dalmais et al., 2011). However, the AA7 genes detected to be upregulated in fruit infection here are not known members of the botcinic acid pathway, suggesting that *B. cinerea* may produce additional uncharacterized polyketide mycotoxins during fruit infection. Even though upregulated *F. acuminatum* genes involved in toxin production are not annotated as members of the AA7 family, fumonisins are products of polyketide metabolism (Alexander et al., 2009). The observed upregulation of fumonisin biosynthesis related genes (*FacuDN12063c0g1i1*, *FacuDN15813c0g1i1*, and *FacuDN9039c0g1i1*) indicates that *F. acuminatum* also produces polyketide mycotoxins during infection of unripe and ripe tomato fruit. However, we also observed upregulation of biosynthetic genes involved in production of trichothecenes (*FacuDN16662c0g1i1*, *FacuDN7264c0g1i1*, and *FacuDN16121c0g1i1*), which indicates that *F. acuminatum* also relies on other toxins during infection of tomato fruit concordant with the classification of *F. acuminatum* as strong toxin producer (Visconti et al., 1989). Additionally, the AA6 family that appears during RR infections of *F. acuminatum* and *R. stolonifer* may be involved in metabolism of host defense compounds. These enzymes are 1,4-benzoquinone reductases, which have been shown to function in fungal protection against destructive host-produced quinones (Jensen et al., 2002; Lee et al., 2007; Gómez-Toribio et al., 2009).

Another physiological factor which may influence the success of infection is the pH of the pathogen-host interface. As the tomato fruit ripens, the apoplast becomes more acidic (Blanco-Ulate et al., 2016b). Furthermore, *B. cinerea* has been shown to acidify the host environment through the production and secretion of oxalic acid (Williamson et al., 2007). A key enzyme in oxalic acid biosynthesis is *BcOAH1* (*Bcin12g01020*), which encodes oxaloacetate hydrolase (Han et al., 2007). This gene is not upregulated during interaction with tomato fruit in any of the treatments. However, there is significant (*P*_adj_ = 2.9*e*^-22^, log_2_FC = -4.52) downregulation of this gene in RR fruit compared to MG fruit. This suggests that, if *B. cinerea* utilizes oxalic acid to acidify tomato fruit, it does so to a much lesser extent in RR fruit where the pH is already comparatively acidic. In contrast, during infection of Arabidopsis roots, *F. oxysporum* relies on alkalinization via peptides known as rapid alkalinizing factors (RALFs; Masachis et al., 2016). However, a BLAST search of RALF sequences, as was performed to identify fungal RALFs in Thynne et al. (2017), revealed no clear RALF genes in our transcriptome of *F. acuminatum*.

The importance of fruit ripening for the success of fungal infections was confirmed by comparing fungal growth and disease development in fruit from wild-type and a non-ripening mutant after fungal inoculation. Growth and morphology of *B. cinerea*, *F. acuminatum* and *R. stolonifer* on *nor* MG and RR-like tomato fruit was comparable to that on wild-type MG fruit. This result is in agreement with our previous report that *nor* tomato fruit is resistant to *B. cinerea* infections (Cantu et al., 2009). The inability to infect non-ripening tomato fruit highlights the dependency of these fungi on the activation and progression of ripening events (e.g., cell wall disassembly during fruit softening, increased redox state, higher available sugars) that transform the host tissues into a favorable environment for disease development.

Altogether, our results confirm that infection success of the three pathogens *B. cinerea*, *F. acuminatum* and *R. stolonifer* largely depends on fruit ripening stage. This is due to all three pathogens sharing similar lifestyles and necrotrophic infection strategies. However, the capacity to infect different plant tissues differs between the three fungi. *B. cinerea* shows distinct strategies in both ripening stages likely due to its ability to induce susceptibility in the host (Cantu et al., 2009), whereas *R. stolonifer* is active almost exclusively in RR fruit. The ability of *F. acuminatum* to infect both MG and RR fruit may be reflective of its especially wide host range, which includes insects in addition to fruit (Logrieco et al., 1992; Rashid et al., 2016). A summary of infection strategies utilized by the three pathogens during infection of MG and RR tomato fruit is shown in Table 2. Further research on which processes identified are required for successful infection would lead to a greater understanding of fruit-pathogen interactions and, ultimately, strategies for their management.

**Table 2:** Summary of strategies utilized by *Botrytis cinerea*, *Fusarium acuminatum* and *Rhizopus stolonifer* during infection of unripe (MG) and ripe (RR) tomato fruit.

Infection strategies in fruit	*B. cinerea*		*F. acuminatum*		*R. stolonifer*	
	Unripe	Ripe	Unripe	Ripe	Unripe	Ripe
ROS production (e.g., NADPH oxidases, laccases)	✓	✓	✓	✓		
ROS detoxification (e.g., catalases, peroxidases)	✓		✓	✓		✓
Proteolysis (e.g., aspartic or subtilisin-like proteases)			✓	✓		✓
Cell wall degradation (e.g., polygalacturonases, cellulases)	✓	✓	✓	✓		✓
Sugar metabolism (e.g., invertases, glucose oxidase)		✓	✓	✓		✓
Toxin production (e.g., polyketides, trichothecenes)	✓	✓	✓	✓		
pH alteration (e.g., oxalic acid production)	✓					


## Data Availability

The datasets generated for this study can be found in GenBank and Gene Expression Omnibus (GEO) database, GGXD00000000, GGWM00000000, and GSE122555.

## Author Contributions

BB-U conceived the original research plan. SP, CS, and BB-U designed the experiments. SP and CS analyzed and interpreted the data. FF-A provided guidance and assistance for fungal growth, identification, and inoculations. SP, SM-P, KG, and CvdA did the fungal inoculations, microscopy, and tissue processing. CS, SM-P, and CvdA extracted RNA and performed the RNA-seq experiments. SM-P, KG, and VP did the RT-qPCR experiments. CS and VP performed the bioinformatic analyses. SP and CS wrote the article with contributions of SM-P, FF-A, SK, and BB-U.

## Conflict of Interest Statement

The authors declare that the research was conducted in the absence of any commercial or financial relationships that could be construed as a potential conflict of interest. The reviewer CC declared a shared affiliation, though no other collaboration, with several of the authors SP, CS, SM-P, CvdA, VP, SK, and BB-U to the handling Editor.

## Footnotes

1 https://github.com/najoshi/sickle

2 https://github.com/vsbuffalo/scythe

3 https://www.bioinformatics.babraham.ac.uk/projects/fastqc/

4 http://solgenomics.net

5 http://www.tcdb.org/

6 http://www.phi-base.org/

7 http://csbl.bmb.uga.edu/dbCAN/

8 http://peroxidase.riceblast.snu.ac.kr/

9 https://github.com/vsbuffalo/sam2counts/

10 http://www.ncbi.nlm.nih.gov/geo
