# Supplementary figures and images for: Infection Strategies Deployed by Botrytis cinerea, Fusarium acuminatum, and Rhizopus stolonifer as a Function of Tomato Fruit Ripening Stage
Source: Front Plant Sci. 2019 Mar 1;10:223. doi: 10.3389/fpls.2019.00223 (PMC6405687; doi:10.3389/fpls.2019.00223)

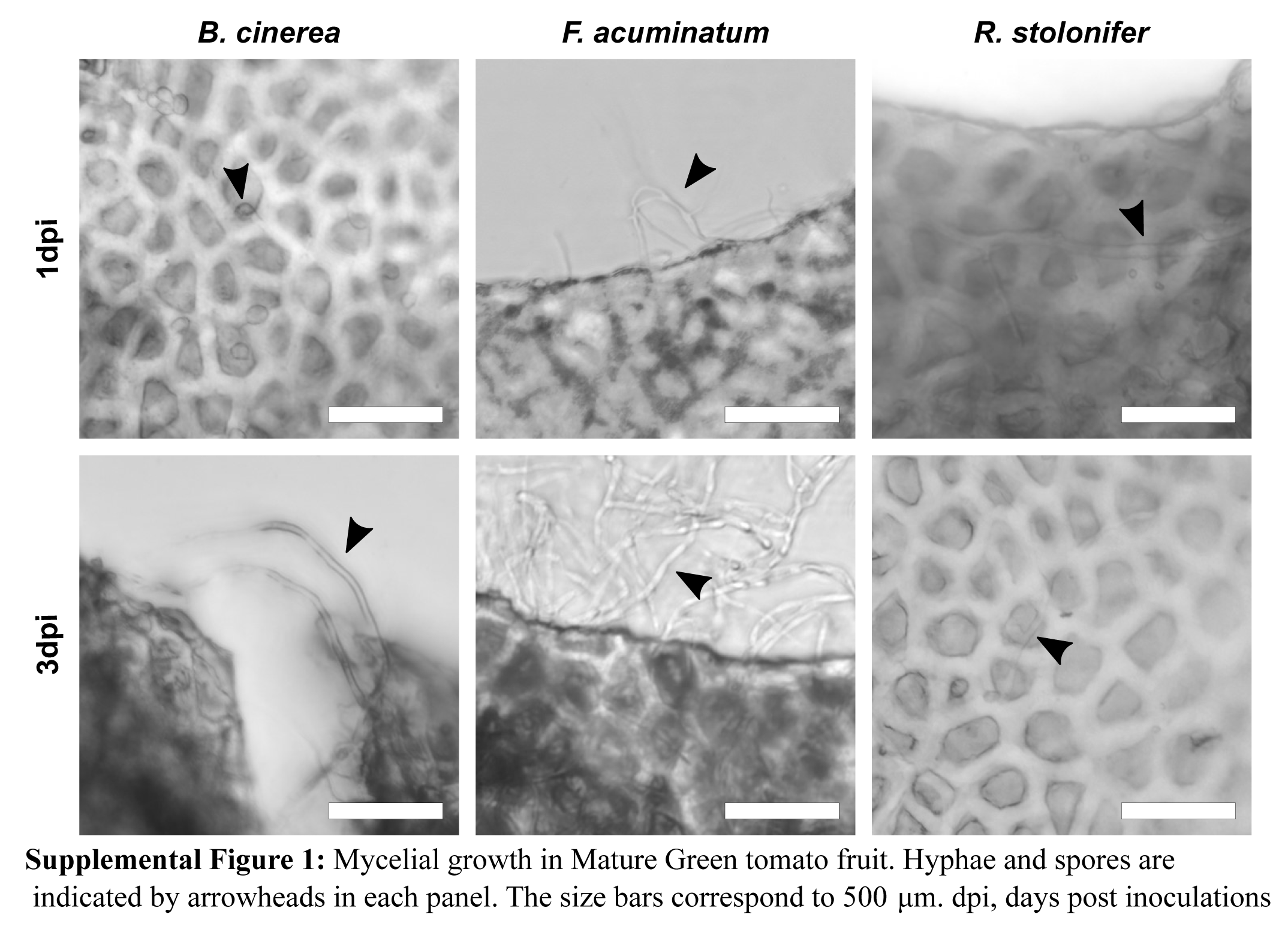

Supplement: Supplementary file 10 [file Image_1.TIF]

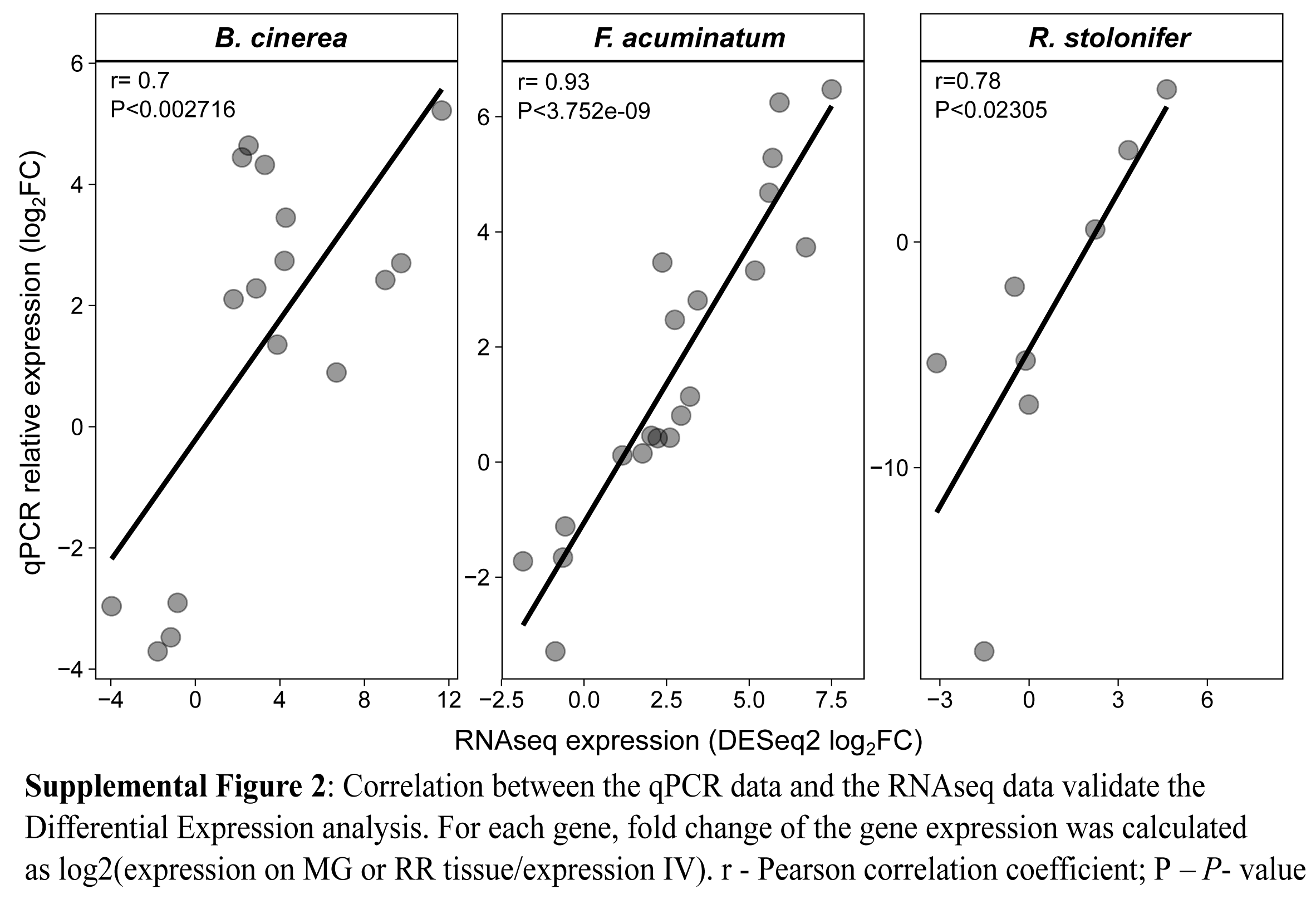

Supplement: Supplementary file 11 [file Image_2.TIF]

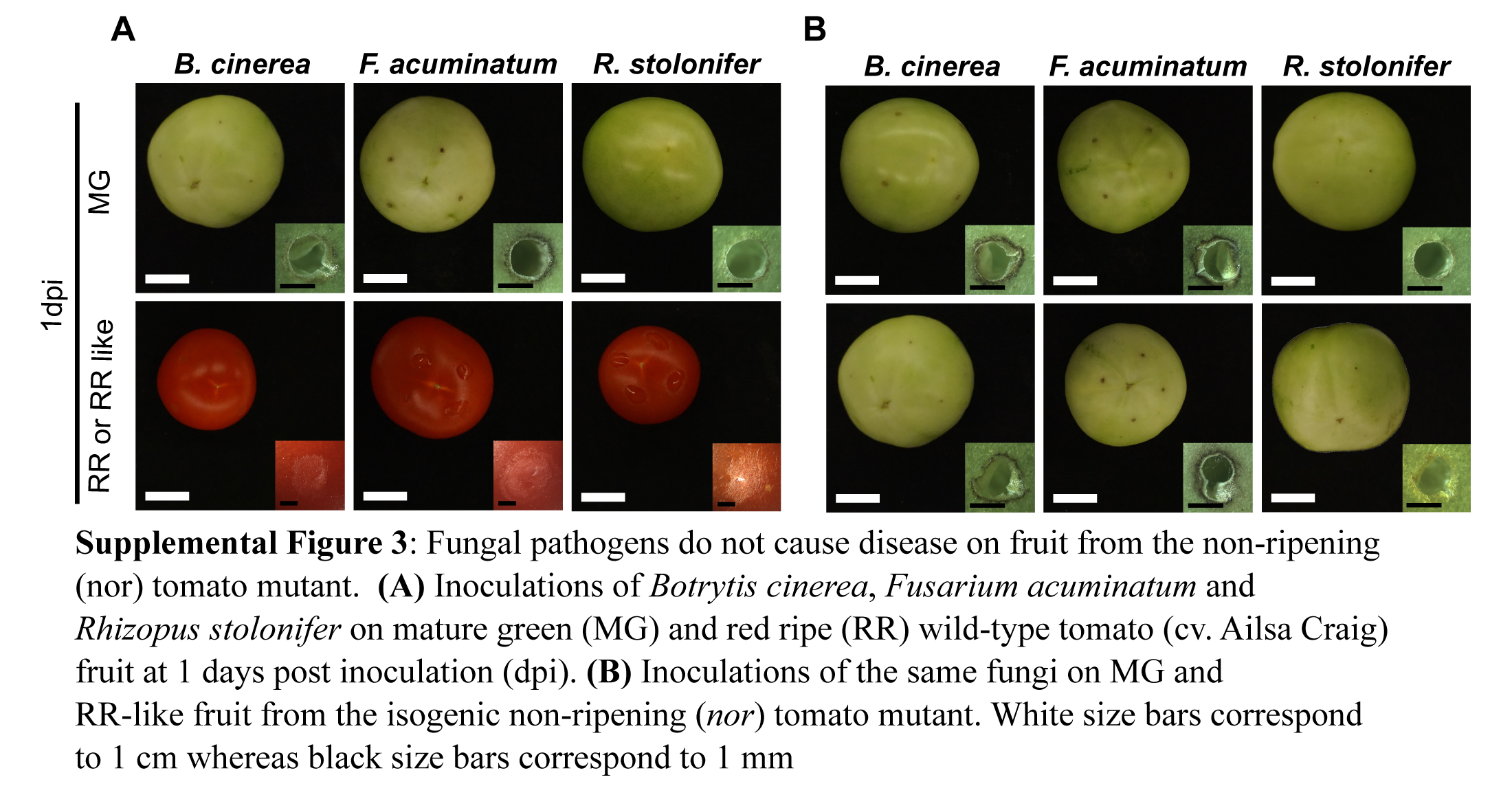

Supplement: Supplementary file 12 [file Image_3.TIF]
